# Rapid gene isolation in barley and wheat by mutant chromosome sequencing

**DOI:** 10.1186/s13059-016-1082-1

**Published:** 2016-10-31

**Authors:** Javier Sánchez-Martín, Burkhard Steuernagel, Sreya Ghosh, Gerhard Herren, Severine Hurni, Nikolai Adamski, Jan Vrána, Marie Kubaláková, Simon G. Krattinger, Thomas Wicker, Jaroslav Doležel, Beat Keller, Brande B. H. Wulff

**Affiliations:** 1 Department of Plant and Microbial Biology, University of Zürich, Zollikerstrasse 107, Zürich, CH-8008 Switzerland; 2 John Innes Centre, Norwich Research Park, Norwich, NR4 7UH UK; 3 Institute of Experimental Botany, Centre of the Region Haná for Biotechnological and Agricultural Research, Šlechtitelů 31, Olomouc, CZ-78371 Czech Republic

**Keywords:** MutChromSeq, Gene cloning, Mutational genomics, Chromosome flow sorting, Triticeae, Wheat, Barley

## Abstract

Identification of causal mutations in barley and wheat is hampered by their large genomes and suppressed recombination. To overcome these obstacles, we have developed MutChromSeq, a complexity reduction approach based on flow sorting and sequencing of mutant chromosomes, to identify induced mutations by comparison to parental chromosomes. We apply MutChromSeq to six mutants each of the barley *Eceriferum-q* gene and the wheat *Pm2* genes. This approach unambiguously identified single candidate genes that were verified by Sanger sequencing of additional mutants. MutChromSeq enables reference-free forward genetics in barley and wheat, thus opening up their pan-genomes to functional genomics.

**Electronic supplementary material:**

The online version of this article (doi:10.1186/s13059-016-1082-1) contains supplementary material, which is available to authorized users.

## Background

The extraordinary success of wheat and barley as major worldwide crops is underpinned by their adaptability to diverse environments, high yield and nutritional content. Identification and manipulation of the genes controlling these traits will help to sustainably increase yields and ensure global food security. However, the large genomes of barley (5.5 Gb) and wheat (17 Gb) coupled with extensive regions of suppressed recombination [1, 2] make traditional map-based gene isolation in these crops both time-consuming and costly. In plants with small genomes, such as *Arabidopsis* and rice, whole genome sequencing on mapping populations has proven a powerful approach to aid gene isolation [3, 4]. Furthermore, whole genome sequencing and comparison of multiple independently derived mutants belonging to the same complementation group permits direct gene identification with little or no requirement for recombination [5, 6]. Because of the genome size of barley and wheat, sequencing whole genomes in multiple mutants is not practical due to the cost and the difficulty of comparison and interpretation of very large datasets. In allopolyploid wheat, this analysis is further complicated by the presence of homoeologues and multiple gene duplications [2, 7].

Various approaches can be used to reduce the sequence complexity of the barley and wheat genomes (Table 1). Exome capture sequencing (ExomeSeq) has been used in barley [8] and wheat [9, 10] to define induced mutations, for forward genetics to define a candidate for the barley *HvPHYTOCHROME C* gene [11] and to clone the barley *many noded dwarf* gene [12] and the wheat stem rust resistance genes *Sr22* and *Sr45* [13]. An exome capture design, however, is biased by only incorporating the known gene space annotated in reference genomes and therefore risks missing out on genes present in the species’ pan genome. A case in hand concerns the pleiotropic resistance gene *Yr36*, a START kinase, which is absent in post Green Revolution wheat [14]. Transcriptome sequencing (RNAseq) can overcome some of these limitations but is biased by the tissue sampled, the time of sampling and the sequencing depth. Also, assembly of RNAseq data from polyploid wheat is problematic due to the co-expression of near-sequence identical homoeologues and RNAseq will only directly reveal mutations in transcribed sequences (while regulatory sequences will be overlooked). Therefore, ExomeSeq and RNAseq have important limitations for isolating genes underpinning adaptive diversity in non-reference accessions.

**Table 1:** Complexity reduction approaches to cloning-by-sequencing in barley and wheat

Complexity reduction	Technology	Sequence costs	Sequence bias	Data handling
Chromosome flow sorting	Complex but optimised for >25 plant species, including barley and wheat	Medium	None	Large data set
Exome capture	Available in several labs	Low	Dependent on reference gene annotation	Small data set
Transcriptome	Simple (depending on tissue sample)	Medium (expression level dependent)	Target gene has to be expressed in sample; mutations must be in transcript	Large data set; de novo assembly problematic in polyploid wheat
None	Simple	Very high	None	Very large data set

**Fig. 1:**
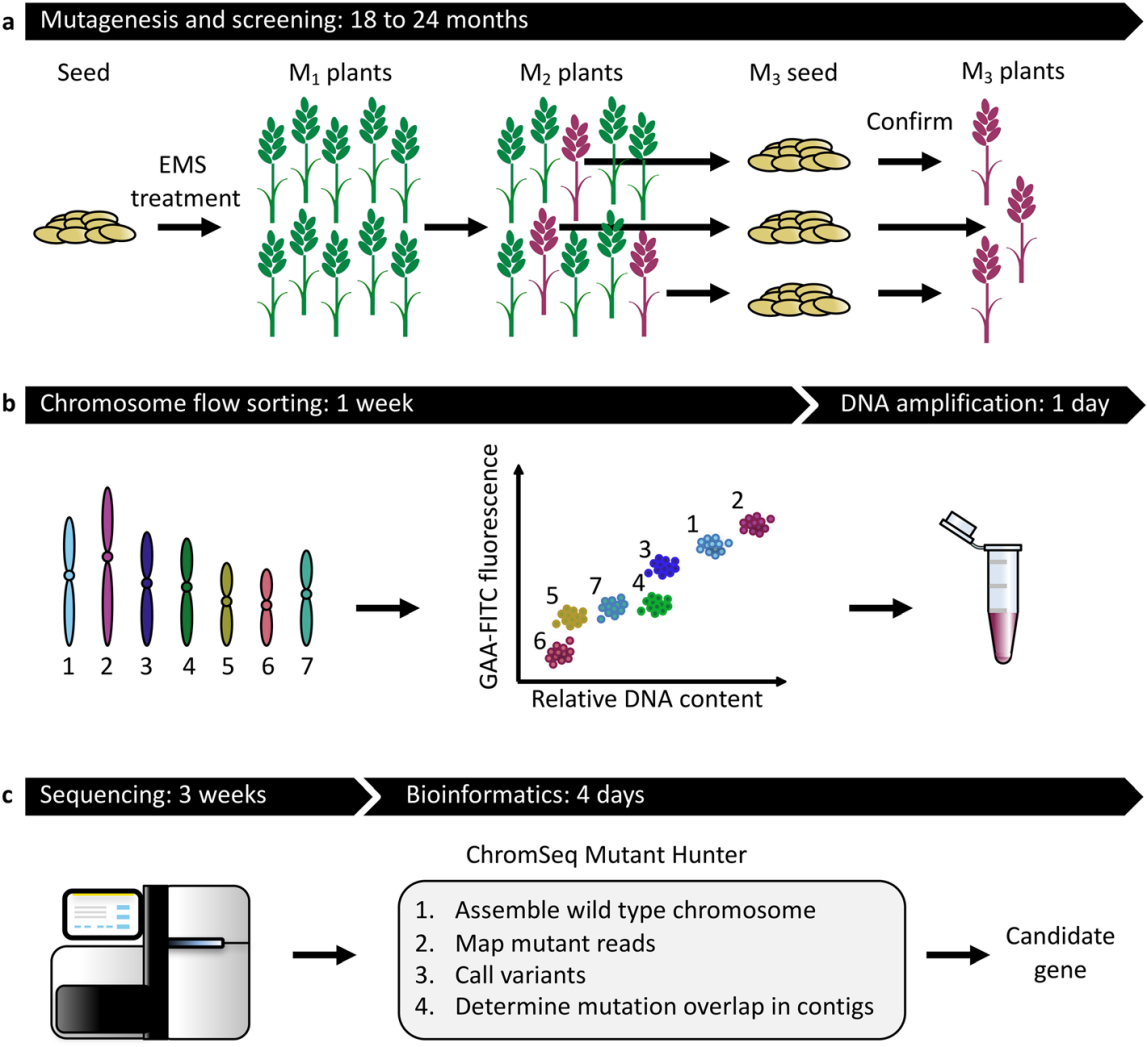
Germplasm structuring and gene isolation by MutChromSeq. **a** Mutagenesis of wheat seed with ethyl methanesulfonate (*EMS*), screening for mutant plants in the M_2_ generation and progeny testing in the M_3_ generation. Additional genetics may be required for some traits to determine complementation groups. **b** Preparation of liquid suspensions of mitotic chromosomes from M_3_ roots and labelling of chromosomes with fluorescently labelled DNA probes followed by flow sorting based on DNA content and fluorochrome signal. Pure chromosome preparations are amplified by phi DNA polymerase. **﻿c** Sequencing of wild type and mutant chromosomes, sequence comparison, and candidate gene identific﻿ation

Chromosome flow sorting and sequencing (ChromSeq) represents a powerful, lossless and sequence-unbiased approach to genome complexity reduction [7]. Recent advances in labelling repetitive DNA on chromosomes prior to flow cytometric chromosome analysis allow purification of the seven barley and 21 bread wheat chromosomes independent of the cultivar [15, 16]. We reasoned that the sequence comparison of multiple independently derived mutant flow sorted chromosomes (MutChromSeq) would allow the identification of induced, causal mutations without the need for positional fine mapping (Fig. 1).

## Results

**Fig. 2:**
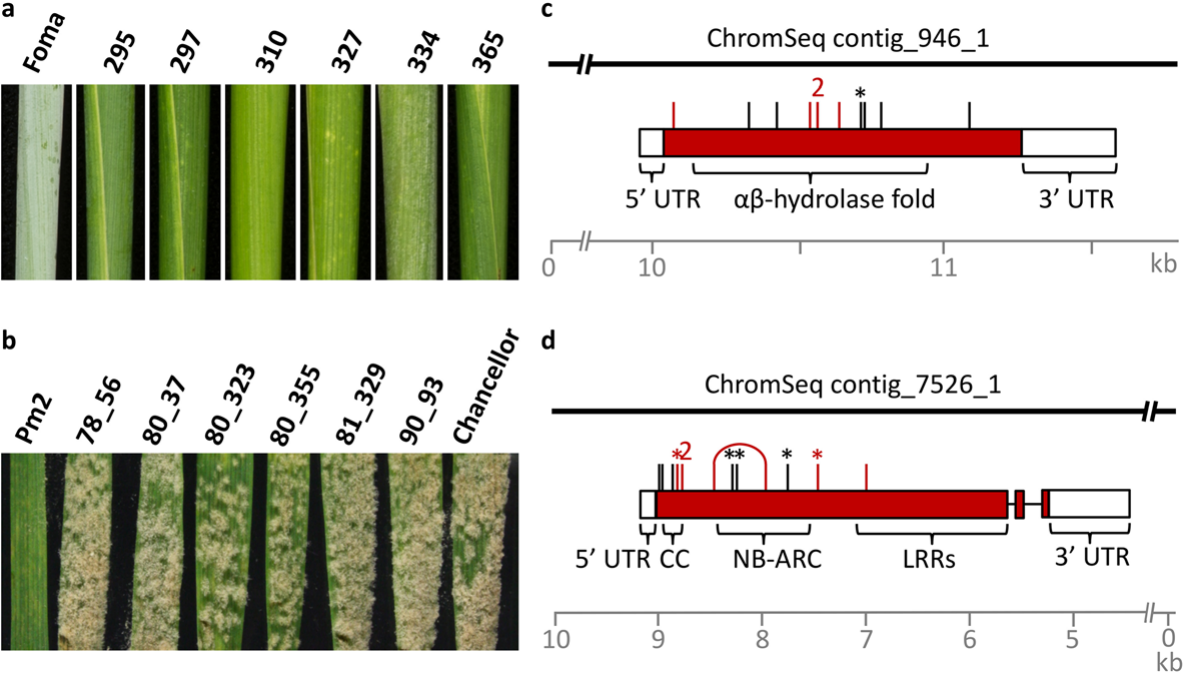
Cloning of the barley *Eceriferum-q* and the wheat *Pm2* genes by MutChromSeq. **a** Wax covered leaf sheath of wild-type barley cultivar Foma and six *eceriferum* (not bearing wax) mutants. Line 334 was deemed to be a cultivar contaminant following sequence analysis. **b** Powdery mildew infected wheat leaves of line CI12632/8*Cc (*Pm2*), six EMS-derived susceptible mutants (*pm2*) and the susceptible control cultivar Chancellor. **c**, **d** The *Eceriferum* (**c**) and *Pm2* (**d**) loci showing intron–exon boundaries, protein domains and 5′ and 3′ untranslated regions (*UTR*). Mutations identified by MutChromSeq are indicated by *red vertical lines*, while mutations identified by Sanger sequencing of additional mutants are indicated by *black vertical lines*. A *number above the line* indicates identical mutations occurring in independent lines. Non-sense mutations are indicated by *asterisks*. Two mutations identified in the *pm2* mutant *80_355* are joined by an *arched line*. CC, coiled-coil; NB-ARC, nucleotide-binding adaptor shared by APAF-1, R proteins, and CED-4; LRR, leucine-rich repeat

As a proof of concept we initially tested MutChromSeq on six ethyl methanesulphonate (EMS)-derived mutants of the recently cloned barley *Eceriferum-q* gene required for epicuticular aliphatic wax accumulation [17, 18] (Fig. 2a; Additional file 1: Figure S1 and Table S1). Flow sorting of chromosome 2H, to which *Eceriferum-q* has been assigned [18–20], followed by multiple displacement amplification (MDA) [21] yielded chromosome DNA preps of high purity (88 to 98 %), quantity (6.6 to 8.2 μg) and molecular weight (3 to 20 kb) (Additional file 1: Table S1 and Figure S2a). We sequenced the amplified chromosomal DNA using Illumina short read sequencing-by-synthesis technology (Additional file 1: Table S2) and performed a de novo assembly of the wild-type cultivar Foma. This resulted in 405,419 contiguous genomic sequences (contigs) of more than 500 bp, totalling 598 Mb with an N50 of 1.4 kb (Additional file 1: Table S3). We then compared the sequence reads from the six mutants to the wild-type assembly and looked at the mutation overlap. We found 57 contigs with single nucleotide variants (SNVs) in three of the mutants, five contigs with SNVs in four mutants and a single 12-kb contig with SNVs in five of the six mutants (Table 2). Closer inspection of the mutant line *eceriferum-q.334* that did not have a mutation in our candidate contig revealed a SNV density of 1 in 1580 bp, whereas the frequency in the other mutants ranged from 1 per 380 kb to 1 per 741 kb (Additional file 1: Table S4). On this basis we concluded that the line *eceriferum-q.334* was a cultivar contaminant and it was excluded from further analysis.

**Table 2:** Mutation overlap in contigs from flow sorted chromosomes of barley 2H and wheat 5D

	Barley 2H (*Eceriferum-q*)	Wheat 5D (*Pm2*)
Number of contigs mutated in 0 lines	197,096	74,260
Number of contigs mutated in 1 line	6,603	2,308
Number of contigs mutated in 2 lines	627	466
Number of contigs mutated in 3 lines	57	80
Number of contigs mutated in 4 lines	5	15
Number of contigs mutated in 5 lines	1	5
Number of contigs mutated in 6 lines	0	2

Sequence homology (BLAST) search of the 12-kb candidate contig against publicly available barley resources [22] and analysis of whole transcriptome reads obtained from wild-type leaf sheath tissue identified a single transcriptional unit supported by one full-length barley cDNA and RNAseq reads (Additional file 1: Figure S2b) with 100 % identity to the recently cloned *Eceriferum-q* gene [18]. The intronless gene spans 1229 bp from the start to the stop codons and contains 5′ and 3′ UTRs of 94 and 331 bp, respectively (Fig. 2c). We confirmed the five mutations identified by MutChromSeq by PCR amplification and Sanger sequencing and sequenced an additional six EMS-derived *Eceriferum-q* mutants and identified another six mutations. All the EMS-derived mutations are G/C-to-A/T transitions that cause nonsense (1) or missense (10) mutations (Additional file 1: Table S5). Based on the EMS canonical mutation frequency and the GC distribution observed in the five EMS-treated and sequenced Foma 2H chromosome contigs, we calculated the probability for a mutation to occur in the same contig in 11 EMS-treated individuals by chance alone to be 1 in 4 × 10^14^ (see “Methods”). In conclusion, these data provide compelling evidence for successful gene identification by MutChromSeq in barley.

To further put MutChromSeq to the test we decided to target a plant *resistance* (*R*) gene in hexaploid wheat. *R* genes typically encode nucleotide binding and leucine-rich repeat containing proteins (NLRs), belong to large multigene families, reside in complex gene clusters with multiple sequence-related paralogues and display extreme sequence and copy number variation between accessions [23]. This often obscures orthogonal relationships and makes functional dissection of *R* gene clusters by recombination alone impractical. We obtained 12 EMS-derived susceptible mutants of the dominant powdery mildew resistance gene 2 (*Pm2*; Additional file 1: Table S5), which has been mapped to chromosome 5D and originally derives from the Ulka donor variety [24]. We chose six of the *Pm2* mutants and the wild-type parent for MutChromSeq (Fig. 2b) and obtained highly pure chromosome samples, which were sequenced on the Illumina platform (Additional file 1: Tables S1 and S2).

Applying the same analysis pipeline we used before, we identified two candidate contigs >1 kb. One could be discarded after manual inspection due to the very large number of SNVs observed between this contig and the mapped wild-type reads indicative of an assembly artefact (Additional file 1: Figure S3), while the other contig contained a full-length NLR type gene encoding domains with homology to a coiled coil, a nucleotide binding site and leucine-rich repeats (Fig. 2d). We confirmed the nature of this short read-assembled 10-kb contig by long-range PCR amplification and sequencing of the complete coding region (Additional file 1: Figure S2d). We converted the contig into a PCR molecular marker and showed that it genetically co-localises with *Pm2*-mediated resistance to a 1.3-cM interval on chromosome 5D (Additional file 1: Figure S2e). Further pivotal confirmation that this gene is *Pm2* was obtained by Sanger sequencing of the remaining six mutants (Additional file 1: Table S5). All mutants were found to contain G/C-to-A/T transitions typical of EMS, which gave rise to predicted nonsense (5) and missense (7) mutations. No other mutations were found in the *Pm2* 10-kb short read-assembled contig in the six chromosome sequenced mutants. Based on the EMS mutation frequency and GC distribution in the six sequenced Pm2 chromosome contigs, we calculated that the probability of getting 12 mutations in the same gene in 12 *pm2* mutants by chance alone to be 1 in 3 × 10^11^ (see “Methods”).

## Discussion

We have used MutChromSeq to successfully reclone the barley *Eceriferum-q* gene and clone de novo the wheat *Pm2* gene. Prior to chromosome flow sorting the target gene must be assigned to a chromosome. This usually requires the phenotypic and genotypic analysis of progeny derived from a sexual cross to allow for the independent assortment of chromosomes. However, recombination per se is not required. This makes the method particularly attractive for cloning genes in recombination-sparse regions. Indeed, the method does not require fine mapping or the construction of a physical reference sequence across a map interval. The approach is fast and robust and in the *Eceriferum-q* example allowed the identification of the correct gene despite the inclusion of a false positive. Our ensuing mapping data and the sequencing of multiple additional independent mutants support the validity and accuracy of MutChromSeq as a powerful approach for gene isolation.

Chromosome flow sorting is a technically demanding procedure requiring expertise in cytogenetics, flow cytometry and molecular biology and the availability of a flow sorter [15, 16]. A group aiming to clone a small number of genes is unlikely to justify the effort required to establish a chromosome sorting pipeline. However, the successful projects on draft genome sequencing in barley [25, 26], rye [27] and wheat [7] demonstrate the viability of collaborative projects.

In the MutChromSeq approach, amplified chromosomal DNA is sequenced. Only tens of thousands of copies of sorted chromosomes are required. These quantities can be purified in less than one day. Thus, we believe that only a small number of specialised laboratories can provide sufficient capacity to satisfy the community demand for chromosome sorting from agronomically important species with large genomes for which protocols for chromosome sorting are available.

Unlike methylation filtration [28], high-C_0_ *t* fractionation [29] or duplex-specific nuclease digestion [30], complexity reduction by chromosome sorting is lossless and all sequences from a particular chromosome are sequenced. Apart from reducing the complexity of the DNA sample to be sequenced, an important advantage of the targeted approach is that it simplifies DNA sequence analysis by avoiding homoeologues in polyploids (wheat) and paralogues and pseudogenes present on other chromosomes (all species).

We recently used an NLR exome capture to clone two resistance genes in wheat by sequence comparison of multiple independently derived EMS mutants [13]. However, not all *R* genes are NLRs. For example, pleiotropic adult plant resistance (APR) genes in wheat encode proteins belonging to disparate structural classes, including an ABC transporter (*Lr34*), a START kinase (*Yr36*) and a hexose transporter (*Lr67*) [14, 31, 32]. Furthermore, genes conferring resistance to necrotrophs, hemibiotrophs and pathogens with an apoplastic lifestyle are less likely to be encoded by NLRs. MutChromSeq avoids the potential problems in gene cloning due to the bias introduced by exome-based capture approaches, including the absence of some genes in reference genome assemblies. Furthermore, compared to ExomeSeq and RNAseq, MutChromSeq is more likely to directly identify mutations in regulatory regions and assemble into the same contig exons separated by large introns. The bridging of exons separated by large introns and identification of native regulatory elements may also be achieved by combining exome capture with single molecule long read sequencing technology [33]. However, long-read technology would at present be expensive to apply to multiple, complete wheat exomes.

## Conclusions

We propose MutChromSeq, a gene cloning method which does not rely on recombination or fine-mapping. Our approach combines mutagenesis, genome complexity reduction by chromosome flow sorting (which is established for a number of plants, including members of the Triticeae) and high-throughput sequencing. Thus, the requirements for our method to work are that the plant species is amenable to mutagenesis, that the target gene can be associated with a clear phenotype and knowledge of which chromosome the gene is on. For genes fulfilling these prerequisites, we offer a fast and inexpensive method that opens up the possibility to clone a large number of previously intractable genes.

## Methods

The aim of this study was to develop a gene cloning method in wheat and barley that would overcome the challenges imposed by their large genomes and extensive regions of suppressed recombination. To this end we combined mutagenesis, which is independent of recombination, with chromosome flow sorting and sequencing. By only sequencing the chromosome a gene has been rough-mapped to, we obtained 21-fold and sevenfold reductions in complexity in wheat and barley, respectively. Comparing the chromosome sequence of multiple mutants allowed the rapid identification of a single candidate gene.

### Chromosome flow sorting and preparation of amplified chromosomal DNA

Liquid suspensions of mitotic metaphase chromosomes were prepared from synchronised root tips of barley [34] and wheat seedlings [35]. GAA microsatellites on chromosomes in suspension were labelled with a fluorescein isothiocyanate (FITC) conjugate [16] and chromosomal DNA was stained by 4′,6-diamidino-2-phenylindole (DAPI) at 2 μg/ml. The chromosome samples were analysed at a rate of 2000 chromosomes/s by a FACSAria SORP (BD Biosciences, San Jose, CA, USA) and sort windows were set up on FITC versus DAPI dot plots to sort chromosome 2H in barley and 5D in wheat. The chromosomes were sorted at rates of 25/s into 0.5-ml PCR tubes containing 40 μl deionised water. Three independent samples of 50,000 chromosomes were sorted from each line and their DNA amplified by multiple displacement amplification [21] using the Illustra GenomiPhi V2 DNA amplification kit (GE Healthcare Life Sciences, Pittsburgh, PA, USA). Amplified DNA samples derived from each line were pooled to achieve higher sequence representation. In order to determine the purity in flow-sorted fractions, during each sort run, 2000 chromosomes were sorted into a 5-μl drop of P5 buffer on a microscope slide and sorted chromosomes were identified by FISH using probes for GAA microsatellites in barley and GAA microsatellites and Afa family repeats in wheat [36].

### lllumina library construction and sequencing

The barley Illumina libraries were constructed following the Broad Institute’s DISCOVAR protocol (http://www.broadinstitute.org/software/discovar/blog/?page_id=375) for PCR free libraries, except New England Biolab reagents were used instead of Kapa. Each mutant library was sequenced on one lane of a HiSeq 2500 with 125 bp paired-end (PE) reads (for each of the mutants) and 250 bp PE reads for wild-type Foma. The wheat libraries were constructed using the Illumina TruSeq protocol with a 250-bp insert size and subjected to 125-bp PE sequencing on an Illumina HiSeq2000 platform. Libraries corresponding to Federation*4/Ulka, Federation and Chancellor were sequenced using independent lanes. In the case of CI12362/8*Cc and the mutants, three libraries were pooled per lane. The average on target chromosome coverage, after correcting for PCR duplicates, ranged from 27 (for the Eceriferum-q.295 line) to 35 (for the Pm2 wild-type line). The details of raw sequence data generated are shown in Additional file 1: Table S2.

### ChromSeq mutant hunter

All raw data were quality trimmed using sickle v.1.2 (https://github.com/najoshi/sickle). We created draft de novo assemblies of chromosome 2H of the wild-type barley cultivar Foma and chromosome 5D of the wild-type wheat line CI12632/8*Cc (Additional file 1: Table S3) using CLC Assembly Cell (https://www.qiagenbioinformatics.com/; version 4.3.0) and standard parameters. The assemblies were masked for repeats using RepeatMasker (http://repeatmasker.org) and the Triticeae Repeat Database (TREP; http://wheat.pw.usda.gov/ITMI/Repeats/) as external library. We then mapped the raw data of the chromosomes from the EMS mutants to the repeat-masked assemblies using BWA [37] v0.7.12. Mappings were filtered for reads not mapping as a pair (samtools view –f2) and PCR duplicates (samtools rmdup) using SAMtools [38] v0.1.19. Mappings were subsequently converted to the mpileup format (samtools mpileup –BQ0). The individual data sets in mpileup format were then integrated to count the number of mutants that had a SNV per contig. Potential SNVs were discarded if the allele frequency was below 99.99 %, the coverage was below 15 and the position was not mutated in more than two mutants. Note, the values above are input parameters to a custom script (http://github.com/steuernb/MutChromSeq). The stringency is dependent on the quality and depth of input data.

### Calculating mutation probabilities

Taking into account that (i) the majority of EMS mutations are G/C-to-A/T transitions [39], (ii) that the majority of our repeat-masked barley and wheat contigs had a similar GC content (Additional file 1: Figure S4) and (iii) that EMS mutations tend to be randomly distributed [40–42], we calculated the probability of a false positive as a function of the length of the contig (Additional file 1: “Supplementary materials and methods” and Table S8). Thus, in barley, the largest probability of a 12-kb contig being mutated in a single mutant was calculated to be 0.05 based on the canonical mutation density observed in *Eceriferum.q-327* (Additional file 1: Tables S4 and S9). Therefore, the probability of the contig being mutated across all 11 mutants by chance alone is (0.05)^11^ or 1 in 4 × 10^14^. Similarly, in wheat, we calculated the largest probability of a 10-kb contig being mutated as 0.11 based on the canonical mutation density observed in mutant *pm2_78.56* (Additional file 1: Tables S4 and S9). Therefore, the probability of the contig being mutated across all 12 *Pm2* mutants is (0.11)^12^ or 1 in 3 × 10^11^.

Further details of materials and methods are shown in Additional file 1: “Supplementary materials and methods”.

## Additional file

Additional file 1: Supplementary material and methods, **Figures S1**–**S4** and **Tables S1**–**S9**. (DOCX 2154 kb)
