# Supplementary material for: Rapid gene isolation in barley and wheat by mutant chromosome sequencing
Source: Genome Biol. 2016 Oct 31;17:221. doi: 10.1186/s13059-016-1082-1 (PMC5087116; doi:10.1186/s13059-016-1082-1)
Supplement: Supplementary file 1 — Supplementary material and methods, Figures S1–S4 and Tables S1–S9. (DOCX 2154 kb) [file 13059_2016_1082_MOESM1_ESM.docx]

**SUPPLEMENTARY INFORMATION MATERIAL AND METHODS**

**Barley germplasm and growth conditions**

All barley germplasm was obtained from the Nordic Genetic Resource Centre (Nordgen). The *eceriferum-q* mutants that we sequenced were generated through the 1950s to 1970s[1, 2] (Supplementary Tables 4 and 5). No backcrossing was performed before chromosome flow sorting and sequencing. Phenotyping was confirmed on adult plants. Seeds were germinated on filter paper at 4°C in the dark and after one week transferred to soil. Plants were grown in individual pots under long day conditions (16 h light, 8 h dark).

**Barley RNAseq**

Barley RNA was extracted from leaf sheath tissue from plants at the GS41 stage (when the flag leaf sheath was extended) using a Direct-zol™ RNA MiniPrep Plus with TRI-Reagent® (Zymo Research). A TruSeq RNA library was prepared and sequenced by The Genome Analysis Centre, UK.

**Characterisation of barley *Eceriferum-q* locus**

The *Eceriferum-q* gene was localised to contig_946_1, which spans 12,503 bp. The gene is on the forward strand from position 10,035 to position 11,264. According to publicly available transcriptome data a full-length cDNA from cultivar Haruna Nijo (accession: EMBL|AK374859) and publicly available gene annotation[3] (MLOC_13397.1) *Eceriferum-q* is a single exon gene (Supplementary Figure 2b). The coding sequence has 99.8% identity at the nucleotide level and 100% identity at the protein level to the orthologuous gene in cultivar Morex (MLOC_13397.1), which was annotated as a “Gibberellin receptor GID1L2”[4]. According to the Barley Genome Explorer[5], it is expressed in developing tillers at six-leaf stage and in developing grain at five days post anthesis. The Haruna Nijo full length cDNA was extracted from early flowering stage.

**Sanger sequencing of barley *eceriferum-q* mutants**

DNA was extracted from the candidates as previously described in Pallotta et al.[6] Genomic DNA from all *eceriferum-q* mutants gave a positive result for PCR amplification with a 426 bp region in the control gene *ELONGATION FACTOR-1α*. A 1,743 bp region, which includes the predicted gene sequence of *Eceriferum.q* and ~250 bp of upstream and downstream sequences, was amplified from the DNA templates using the Eceriferum.q_amp Forward and Reverse primers (Supplementary Table 7). The purified amplicons were Sanger sequenced by GATC Biotech. The forward strand was sequenced twice in two different overlapping regions using the primers Eceriferum.q_1_Forward and Eceriferum.q_2_Forward, and the reverse strand was sequenced twice in two overlapping regions using the primers Eceriferum.q_1_Reverse and Eceriferum.q_2_Reverse (Supplementary Table 7).

**Wheat germplasm and growth conditions**

Two susceptible wheat cultivars Federation (GRIN accession number CItr47341; with pedigree Purplestraw 14A/Yandilla) and Chancellor (Cc) (GRIN accession number CItr1233; with the pedigree Carina/Mediterranean//Dietz/Carina/3/P1068/3*Purplestraw), and their near-isogenic lines (NILs), Federation*4/Ulka (derived from Ulka BC_3_ to Federation) and CI12632/8*Cc (derived from CItr1532 BC_7_ to Chancellor) carrying the *Pm2* resistance gene[7], were used in the present study. The original donors for powdery mildew resistance, Ulka[8] (GRIN accession number CItr2898; with unknown pedigree) and Allard No 52-1-1-17-1 (GRIN accession number CI12632[9]; with the pedigree CI15232/timopheevii), although of different origin, were reported to have one common dominant gene conferring resistance to powdery mildew[10], which later was designated as *Pm2[7]*. Here we have extended this analysis by testing CI12632/8*Cc and Federation*4/Ulka with 32 additional powdery mildew isolates in repeated infection tests and the lines were not differentiated by any of the isolates (Supplementary Table 6). Two recently reported new alleles of *Pm2*, temporarily named *Pm2b* and *Pm2c*, have been described at the *Pm2* locus[11, 12]. Thus, the *Pm2* allele cloned in this study will be redesignated as *Pm2a* in the near future.

Plants were grown in Jiffy plots (4×5 cm hole, Jiffy A/S, Denmark) in plastic trays (each tray contained 96 Jiffy plots) in a growth chamber cycled at 20°C/16°C, 16/8h photoperiod with 80% relative humidity. DNA isolation was performed as previously described[13].

**Leaf segment infection test with the powdery mildew isolate *94202***

Isolate *94202*, collected in Switzerland in the year 1994 was used for the infection tests because of its avirulence/virulence pattern: avirulent (no visible symptoms observed) on the *Pm2* lines, and highly virulent (leaves fully covered by mycelia) on the susceptible lines.

For the infection tests we used fully expanded first leaves of 11-day-old plants grown under the conditions mentioned above. Leaf segments were placed with their adaxial side up on 0.5% phytoagar (30 ppm benzimidazole) in Petri dishes. Infections were done with freshly propagated conidiospores[14] using 5 ml Pasteur glass pipettes and a settling tower[15]. Plates were then transferred to an incubator set at 20°C, 80% relative humidity with 16 h light/8 h dark cycle with 50 *µ*mol m^-2^s^-1^ photon flux density. Disease levels were assessed 7-9 d after inoculation as previously described[16], using a discrete quantitative percentage scale from 0% indicating no visible disease symptoms to 100% indicating complete coverage of the leaf with sporulating colonies.

**Genetic mapping of *Pm2* mediated resistance**

As *Pm2* is a dominant resistance gene, genetic mapping was done based on susceptible progeny of an F_2_ population from a cross between Federation*4/Ulka and Federation, in order to have a maximal information content from phenotyping. The F_2_ population of 340 individuals segregated with a ratio of 260 resistant to 80 susceptible, fitting a single dominant gene segregation ratio of 3:1 (X^2^_3:1_ = 1.48, *p* = 0.22). The leaf segment infection tests were performed as described above.

For the genetic mapping of *Pm2* mediated resistance we developed genotype-specific polymorphic markers based on sequence data of flow-sorted 5D chromosomes of the two genotypes Federation*4/Ulka and Federation. A workflow detailing the process is described in Shatalina et al[17]. Briefly, we used the Federation*4/Ulka *de novo* assembly as a reference on to which Federation reads were mapped for the identification of single nucleotide polymorphisms (SNPs). *De novo* assembly for Federation*4/Ulka after filtering data resulted in 986,549 contigs with an average length of 683 bp and an estimated 57x chromosome coverage. For *de novo* assembly and mapping we used functions “De Novo Assembly” and “Map Reads to Reference” commands with default parameters of the CLC Genomics Workbench 7.5 (CLC, Aarhus, Denmark). 272,876 contigs had at least one SNP. To detect putative genes, we used BLASTN against a *B. distachyon* coding sequences database to select only contigs containing coding sequences of genes. Besides, only contigs with a length of 5,000 bp or longer and a coverage between 5 and 50 were considered for further selection. For the final selection, we only used contigs which displayed a match to Chromosome Survey Sequence (CSS)[18] and/or have their homologs in the 5D chromosome of *Ae. tauschii.* As we did not have the *Pm2* gene flanked, we chose gene-containing contigs expected to be regularly spread across the 5D chromosome based on syntenic location in *Brachypodium* (Bradi4g00200 – Bradi4g07997; Bradi4g38400 – Bradi4g45500) and/or chromosome 5D in *Ae. tauschii.* Ninety-six SNPs were selected for the genetic mapping of the *Pm2* gene on the 80 F_2_ lines phenotyped as susceptible. Genotyping was carried out by LGC Genomics (Hertfordshire, UK) using fluorescence-based competitive allele-specific PCR (KASPar) assay. A total of 45 SNPs were polymorphic between the parents. In addition, we screened markers previously reported to be linked to the *Pm2* gene (*cfd81*, *gwm190* and *cfd18*)[19] as well as markers reported to be close to the *Pm2* locus (*cfd78;* two SCAR markers, SCAR112 and SCAR203; and one EST-derived marker MAG6176[20, 21]). Only the *Pm2*-linked SSR marker *Cfd81[19]* showed polymorphism between the parents.

A preliminary set of 46 polymorphic parents was considered for the mapping of *Pm2*. However, some of these markers cosegregated. Thus, the map was constructed using only one marker from each set of cosegregating markers. A total of 32 polymorphic markers were mapped on the 80 F_2_ susceptible plants using MAPMAKER/Exp (version 3.0b) with a LOD threshold of 3.0 and recombination fractions were converted to map distances (cM) using the Kosambi map function[22]. The genetic linkage map was drawn using MapChart[23] v 2.2. The resulting map is displayed in Supplementary Figure 2e. Only the markers that map closest to *Pm2* are shown.

**Generation, screening and characterization of EMS-induced *Pm2* mutants**

Mutants were generated by treating CI12632/8*Cc seeds with the mutagen ethyl methanesulfonate (EMS) at a concentration of 0.5% for 16 hours. Three thousand CI12632/8*Cc seeds were soaked overnight in ultrapure water for 16 h at 4°C, followed by a treatment in EMS solution at room temperature while shaking at 150 rpm under the above mentioned conditions. Seeds were then washed in tap water.

EMS-treated M_1_ seeds were grown in the field in plots consisting of five 1 m-long rows. One spike per plant was bagged to prevent cross-pollination. At maturity stage, around 200 individual bagged spikes from each plot were harvested and threshed together. EMS M_2_ seeds were individually sown and subjected to powdery mildew infection tests as explained above. From a screen of approximately 6,000 M_2_ seedlings we isolated twenty-eight putative *pm2* mutants.

To discard *pm2* mutants arising due to the loss of the complete chromosome 5D, we amplified the SSR marker *gwm190* that maps to the distal region of the short arm of chromosome 5D[24]. Two of the mutants were discarded as the SSR maker did not amplify on them. The remaining twenty six putative *pm2* mutants, cultivar Chancellor and its *Pm2* near-isogenic line, from which the EMS-derived *pm2* mutants were generated, were subjected to large scale SNP screening by the 15K Infinium wheat chip that contains SNPs from the Illumina iSelect 90k wheat array ([21]; TraitGenetics GmbH, Gatersleben, Germany) to check if they had large deletions which could not be detected with marker *gwm190*. From the original dataset compromising 13,006 SNPs, 99 SNPs failed in all samples, leaving a total of 12,907 scorable SNPs. The vast majority of them could be genotyped in the tested genotypes. The number of SNPs that could not be genotyped on CI12632/8*Cc and Chancellor was 39 and 72, respectively. In the case of the putative *Pm2* mutants it varied from 31 to 197, leading us to conclude that no large deletions were present.

Susceptibility of these twenty-six putative mutants to the wheat powdery mildew pathogen was confirmed in the M_3_ generation based on 10 different M_3_ plants from each M_2_ family. Nine mutants turned out to be resistant to the *Pm2*-avirulent isolate 94202. In addition, none of these nine were subsequently found to have punctual mutations in the full-length sequence of the *Pm2* candidate gene. Out of the seventeen remaining putative *pm2* mutants, six were selected for chromosome flow-sorting and sequencing. No backcrossing was performed before chromosome flow sorting and sequencing. Eleven *pm2* mutants were analysed by Sanger sequencing once the gene had been identified by flow-sorting. Five mutant pairs showed identical punctual mutations, most likely due to the fact they were bulk-harvested and they belonged to the same M_2_ family. For each pair, only one mutant was considered for further analysis.

**Identification of a candidate *Pm2* contig by MutChromSeq**

After the SNP calling of flow-sorted 5D chromosome data from six individual *pm2* mutants on the assembly of the wild type *Pm2* line (CI12632/8*Cc), two candidate contigs >1 kb were found to have a mutation in each mutant line. Only for the contig_7526_1 did all the mutations fall within an open reading frame associated with an NLR-type resistance gene (Supplementary Table 5, Supplementary Figure 3b).

**Genetic mapping of *Pm2* candidate gene ChromSeq contig_7526_1**

SNP markers were designed based on two different SNPs identified between Federation*4/Ulka and Federation in the *Pm2* candidate gene contig_7526_1, supposed to include the *Pm2* candidate gene. Both SNP markers are placed in the 3’UTR region. The two pair of primers used for the genotyping were JS354 x JS355 and JS303 x JS305 (Supplementary Table 7). The SNP genotyping on the F_2_ population was performed using the high-resolution melting (HRM) technique. The real-time PCR reaction (10-μL volume) contained 1 ng of genomic DNA, 5μl of 2x Kapa HRM Fast PCR kit (Kapa Biosystems, Cape Town, South Africa), 1μl of 2.5 mM MgCl_2_ and 0.2 μl of 10 μM of each primer (Sigma-Aldrich). All the reactions were performed in duplicate using the Bio-Rad CFX96 real-time PCR system (Bio-Rad, USA). The amplification conditions were as follows: 95°C for 2 min, then 40 cycles at 95°C for 5 s and 60°C for 30 s. Afterwards, PCR products were heated for 1 min at 95°C and cooled to 60°C for 1 min. After the PCR amplification steps, melting curves for the products were generated based on the temperature ramping and fluorescence acquisition settings recommended by the manufacturer, a temperature ramping from 65 to 95°C, rising by 0.1°C/2 s. The resulting melting curves were analyzed with Bio-Rad Precision Melt Analysis™ software.

**Characterisation of the wheat *Pm2* locus**

The position of the *Pm2* start codon was predicted based on homology to Bradi4g06470, the closest homolog from *Brachypodium*. Intron/exon structure at the 3' end was predicted through alignment with a transcript from *Triticum aestivum* (Genbank accession GAJL01276631, the Genbank record does not specify which *T. aestivum* cultivar was used) (Supplementary Figure 2c). This transcript indicates that the 3' UTR has a size of at least 720 bp. The gene is also completely spanned by a transcript from *T. urartu* (Genbank accession GAKL01040061), which supports the intron/exon structure predicted based on the *T. aestivum* cultivar (Supplementary Figure 2d). However, sequence homology to *T. urartu* decreases in the 3' region. Nevertheless, the *T. urartu* transcript suggests that the 5' UTR is at least 100 bp in length. Within this 5' UTR, no alternative start codons were found and the next upstream in-frame stop codon is found at position 9,232 (in ChromSeq contig_7526_1), just upstream of the end of the *T. urartu* transcript, which makes it very unlikely that an additional intron was missed.

A long range PCR (PCR 1; Supplementary Figure 2c) on genomic DNA of all parents and EMS-derived *Pm2* mutants was performed using the primers JS320 x JS305 followed by a nested PCR with JS314 (with a binding site 244 bp before the start codon), and the reverse primer JS315 (with a primer binding site 3,819 bp 3’ from the start codon). For PCR amplification, the KAPA Hifi HotStart Polymerase (KK2502, Kapa Biosystems) was used with an annealing temperature of 60°C and an extension time of 4:00 and 3:30 min for the full-length and nested PCR, respectively. The PCR products were sequenced with the internal primers JS317, JS321, JS324, JS328, JS330, JS342, and JS345 (Supplementary Table 7).

An additional PCR (PCR 2; Supplementary Figure 2c) with primers JS350 x JS313 was used to amplify the second and third predicted exons for the resistant parent, CI12632/8*Cc, and the PCR products were sequenced with the internal primers JS303 x 305 (Supplementary Table 7).

For amplification and sequencing of the 3’ part of the cDNA of *Pm2,* RNA from CI12632/8*Cc and Chancellor was extracted using the Promega SV Total RNA Isolation System kit (Z3100; Promega, Dübendorf, Switzerland). First strand cDNA was synthesized from 1 µg of total RNA using 2 mM oligo-dT-primer (5’dT20NV-3’), the reverse transcriptase SuperScript III (18080-044, Thermo Fisher) and RNaseOUT Recombinant RNase Inhibitor (10777-019, Thermo Fisher) according to the manufacter’s protocol. In the resistant parent, CI12632/8*Cc, we could confirm *Pm2* transcript accumulation by PCR using JS350 with a binding site 3,612 bp 3’ from the start codon and primer JS313, with a binding site on the 3’ UTR region of the gene. In addition, we could confirm intron/exon structure by sequencing of the PCR product amplified by the primer pair JS350 x 313 (Supplementary Figure 2d).

**Mutation probability calculations**

In order to calculate the probability of obtaining a false positive in our analysis, we considered only G/C-to-A/T transitions, since this is the most dominant form of EMS mutation[25, 26]. We observed that the majority of contigs had a similar GC content (Supplementary Figure 4). Based on this we assumed a random distribution of G/C-to-A/T transitions (canonical mutations), as has previously been observed in plants and animals[27-29]. Therefore, the mutation probability (*P_m_*) becomes a function of mutable contig length (*l*), and mutation density (*M*), defined as:

*P_m_* = *l* · *M*

For any contig in a given mutant, *l* is calculated as number of GC bases in the contig (the mutable length), while *M* is calculated as being equal to (total number of canonical SNPs)/(total number of GC bases). *M* is thus considered constant for all contigs in a given mutant.

This makes the probability of a mutated contig being shared across *x* number of mutants (*P_w_*):

*P_w_* = *P_m_^x^*

We calculated *M* for both assemblies using information from the mutants which had the largest number of canonical SNPs (Supplementary Table 4). We then divided the assembly into contig bins, based on contig length increments of 100 bp, starting at 500 bp contig sizes. We calculated the *P_m_* of a median sized contig in each bin, and multiplied this with the number of contigs in each such bin. Summing up these numbers over all the bins for a given mutant gives us the likely total number of false positives in a single mutant. We similarly calculated the likely number of false positives of any size that would result from comparing 2, 3, 4, 5 and 6 mutants (Supplementary Table 8). All contigs that were less than 500 bp, and/or which had a coverage of less than 15 in the wildtype were removed from both assemblies prior to analysis, since a similar filtering was also applied in the mutant hunter pipeline. Our analysis shows that the number of shared contigs (of any size) between different mutants by chance alone decreases as more mutants are compared.

**
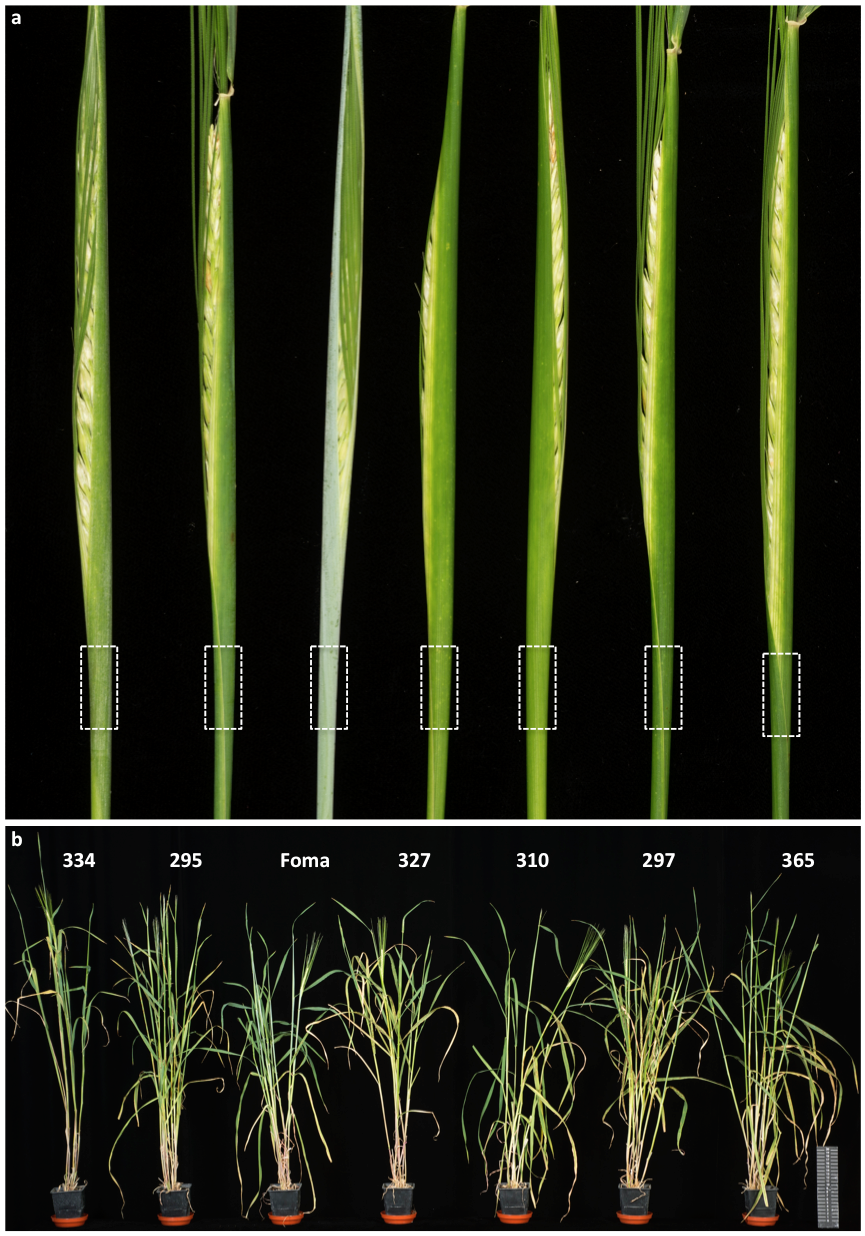
**

**Figure S1**. Phenotype of wild type barley and *eceriferum-q* mutant plants.

Glasshouse grown barley plants (bottom panel) with a close-up of individual spikes just before emergence (top panel), showing wax covered leaf sheath in cultivar Foma, and absence of wax in the six mutants. Note that line 334 was found to be a cultivar contaminant based on an excessively high polymorphism rate when compared to Foma. Stippled rectangles indicate cut-outs shown in Figure 2b.

**
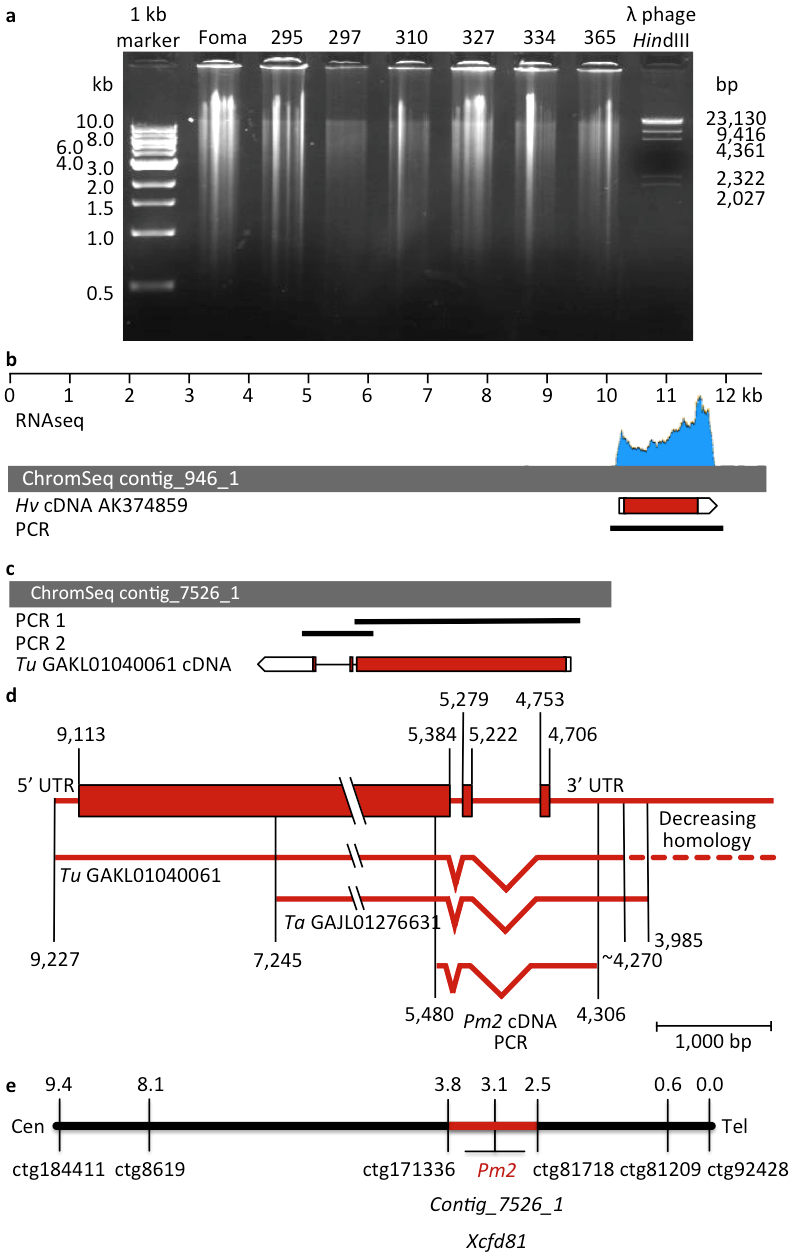
**

**Figure S2**. Flow sorted and multiple displacement amplified DNA and analysis of the barley *Eceriferum-q* and wheat *Pm2* loci.

(a) Approximately 150 to 200 ng of barley chromosome 2H MDA DNA was loaded per well and run in a 0.8% agarose gel.

(b) ChromSeq contig_946_1 (12,503 bp) containing the *Eceriferum-q* candidate gene. RNAseq analysis on leaf sheath tissue (top panel) identified a single transcriptional unit supported by a full-length cDNA (accession: EMBL|AK374859). The ChromSeq contig surrounding the *Eceriferum-q* gene was verified by amplification and sequencing of a 1,753 bp PCR product.

(c) ChromSeq contig_7526_1 (10,210 bp) containing the *Pm2* candidate gene. The structure of the contig around *Pm2* was verified by amplification and sequencing of two overlapping PCR products. The intron/exon structure is supported by a transcript from *Triticum urartu* (Tu GAKL01040061). Scale as in (a).

(d) Detailed analysis and prediction of the *Pm2* transcript and intron/exon structure based on publicly available data and amplification and sequencing of the 3’ part of the *Pm2* cDNA. The predicted *Pm2* transcript is shown in reverse orientation (relative to contig_7526_1) in order to display the gene in forward orientation.

(e) Genetic linkage map of the short arm of chromosome 5D around *Pm2*. The map is based on 80 susceptible F_2_ plants derived from the cross Federation*4/Ulka (Pm2) and Federation. The centiMorgan (cM) position is indicated on the left. Cen and Tel indicate the proximal (centoremeric) and distal (telomeric) ends of the map.

**
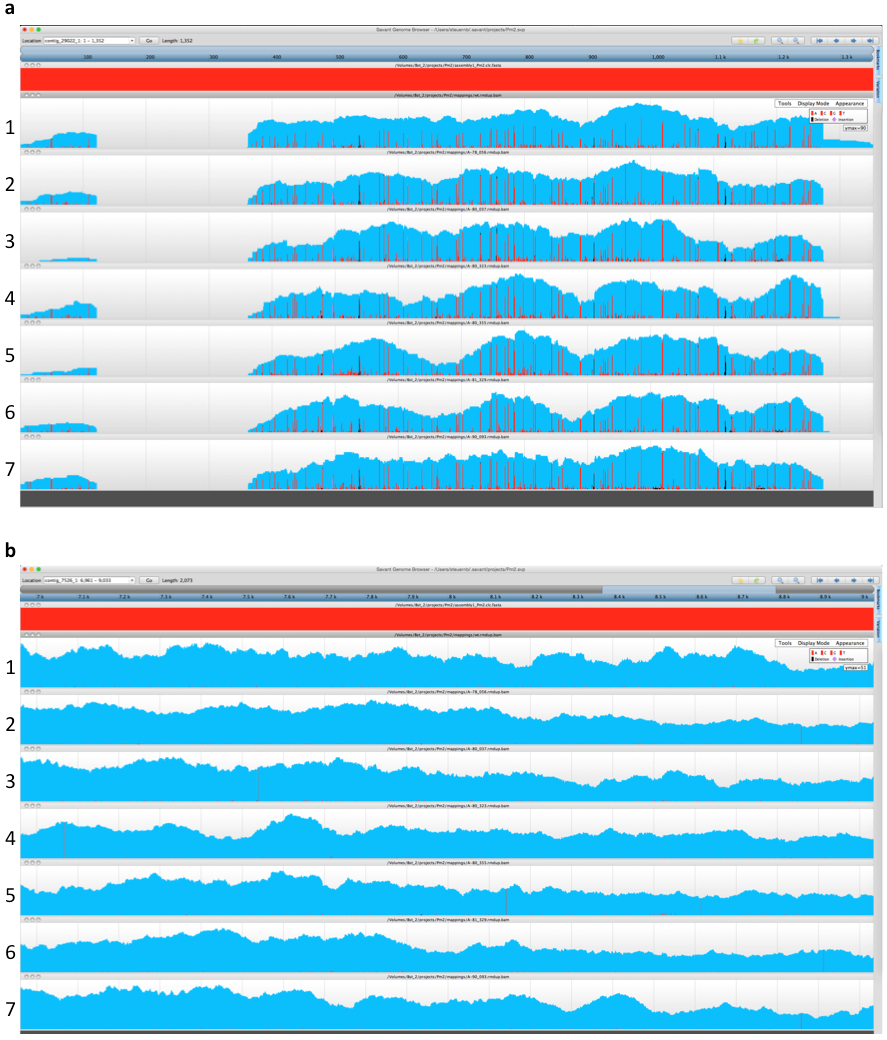
Figure S3**. Candidate *Pm2* contigs.

Visualization in Savant[25] of wild type (track 1) and mutant reads (tracks 2-7) mapped to two candidate *Pm2* contigs. Blue background shows coverage, red shows variations from reference and black shows deletions.

(a) Contig_29022_1 (1,352 bp). The large number of SNVs between wild type raw data and reference indicate that this contig is an assembly artifact.

(b) Region in contig_7526_1 (from 6,961 to 9,033 bp) showing identified causal SNVs.


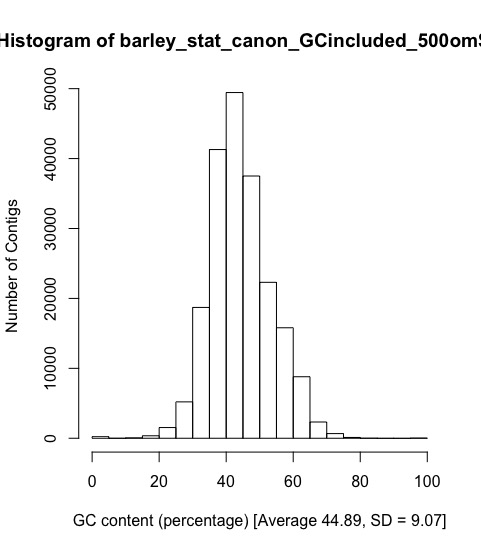

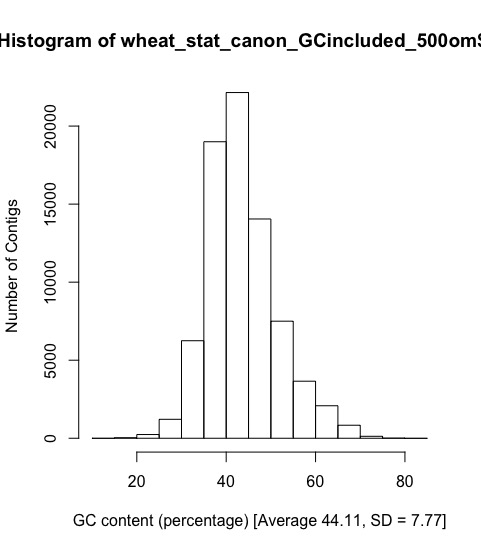
a b

**Figure S4.** GC content in barley and wheat flow sorted chromosome assemblies.

Histogram of GC content (expressed as percentage) in repeat-masked contigs from the (a) Eceriferum-q and (b) Pm2 wildtype assemblies.

**Table S1.** The purity in flow-sorted barley and wheat chromosome fractions and yields of amplified chromosome DNA.

| **Species** | **Cultivar** | **Line** | **Chromosome** | **Purity (%)** | **DNA amount**  **(µg)^1^** |
| --- | --- | --- | --- | --- | --- |
| Barley | Foma | Wild type | 2H | 90.8 | 7.6 |
| Barley | Foma | *eceriferum-q.295* | 2H | 87.8 | 6.6 |
| Barley | Foma | *eceriferum-q.297* | 2H | 97.0 | 7.1 |
| Barley | Foma | *eceriferum-q.310* | 2H | 97.6 | 6.7 |
| Barley | Foma | *eceriferum-q.327* | 2H | 92.4 | 7.6 |
| Barley | Foma | *eceriferum-q.334* | 2H | 98.1 | 8.2 |
| Barley | Foma | *eceriferum-q.365* | 2H | 91.6 | 7.7 |
| Bread wheat | Federation*4/Ulka | Wild type | 5D | 84.0 | 10.0 |
| Bread wheat | Federation | Wild type | 5D | 92.0 | 10.2 |
| Bread wheat | Chancellor | Wild type | 5D | 95.5 | 8.3 |
| Bread wheat | CI12632/8*Cc | Wild type | 5D | 94.6 | 7.3 |
| Bread wheat | CI12632/8*Cc | *pm2_78_56* | 5D | 95.4 | 8.0 |
| Bread wheat | CI12632/8*Cc | *pm2_80_37* | 5D | 94.1 | 7.1 |
| Bread wheat | CI12632/8*Cc | *pm2_80_323* | 5D | 94.7 | 8.2 |
| Bread wheat | CI12632/8*Cc | *pm2_80_355* | 5D | 96.1 | 7.9 |
| Bread wheat | CI12632/8*Cc | *pm2_81_329* | 5D | 90.7 | 7.5 |
| Bread wheat | CI12632/8*Cc | *pm2_90_93* | 5D | 93.8 | 7.7 |

^1^ Following multiple displacement amplification.

**Table S2.** Raw Illumina sequence data generated in this study.

| **Sample** | **Library** | **Reads** | **Read length** | **Output** |
| --- | --- | --- | --- | --- |
| Foma | 1566_LIB18287_LDI15709_NoIndex_L001 | 198,838,474 | 250 | 49.7 |
| *eceriferum-q.295* | 1543_LIB18288_LDI15710_NoIndex_L004 | 355,939,188 | 125 | 44.5 |
| *eceriferum-q.297* | 1567_LIB18289_LDI15711_NoIndex_L001 | 349,062,548 | 125 | 43.6 |
| *eceriferum-q.310* | 1567_LIB18290_LDI15712_NoIndex_L002 | 384,212,174 | 125 | 48.0 |
| *eceriferum-q.327* | 1567_LIB18291_LDI15713_NoIndex_L003 | 387,418,254 | 125 | 48.4 |
| *eceriferum-q.334* | 1567_LIB18292_LDI15714_NoIndex_L004 | 361,011,688 | 125 | 45.1 |
| *eceriferum-q.365* | 1567_LIB18293_LDI15715_NoIndex_L005 | 397,273,056 | 125 | 49.7 |
| Federation*4/Ulka | 20140911.A-FDU_5D | 686,095,344 | 125 | 85.8 |
| Federation | 20140911.A-FD_5D | 530,808,034 | 125 | 66.4 |
| Chancellor | 20150618.A-Chancellor | 67,007,954 | 125 | 8.4 |
| CI12632/8*Cc | 20150821.A-CI | 121,118,242 | 125 | 15.1 |
| *pm2_78.56* | 20150821.A-78_56 | 139,946,006 | 125 | 17.5 |
| *pm2_80.323* | 20150821.A-80_323 | 140,175,724 | 125 | 17.5 |
| *pm2_80.355* | 20150821.A-80_355 | 124,071,696 | 125 | 15.5 |
| *pm2_80.37* | 20150821.A-80_37 | 106,921,678 | 125 | 13.4 |
| *pm2_81.329* | 20150821.A-81_329 | 125,535,336 | 125 | 15.7 |
| *pm2_90.93* | 20150821.A-90_93 | 161,998,502 | 125 | 20.2 |

**Table S3.** ChromSeq assembly statistics.

|  | **Barley cultivar Foma** | **Wheat CI12632/8*Cc (Pm2)** |
| --- | --- | --- |
| Number of contigs^1^ | 781,686 | 320,167 |
| Number of contigs ≥500 bp | 405,419 | 94,594 |
| Number of contigs ≥1,000 bp | 148,822 | 42,448 |
| Sum of all contigs ≥500 bp | 598,391,537 bp | 155,470,444 bp |
| N50 | 1,404 bp | 1,192 bp |

^1^Note that in Table 1, the number of contigs is different because they are based on BLAST to barley and wheat.

**Table S4.** SNV frequency in flow sorted mutant chromosomes.

| **Mutant** | **Canonical SNVs** | **GC bases in wild type assembly** | **Mutation density**  **(SNVs per G or C base)** | **EMS dose** |
| --- | --- | --- | --- | --- |
| *eceriferum-q.295* | 245 | 106,892,814 | 2.29e-06 | 0.12%, 24 h |
| *eceriferum-q.297* | 457 | 106,892,814 | 4.28e-06 | 0.25%, 72 h |
| *eceriferum-q.310* | 511 | 106,892,814 | 4.78e-06 | 0.17%, 24 h |
| *eceriferum-q.327* | 992 | 106,892,814 | 9.28e-06 | 0.25%, 5 h |
| *eceriferum-q.334** | 19,998 | 106,892,814 | 0.000187 | 0.7%, 5 h |
| *eceriferum-q.365* | 307 | 106,892,814 | 2.87e-06 | 0.17%, 5 h |
| *pm2_78.56* | 1265 | 52,061,452 | 2.43e-05 | 0.5%, 16 h |
| *pm2_80.323* | 777 | 52,061,452 | 1.49e-05 | 0.5%, 16 h |
| *pm2_80.355* | 553 | 52,061,452 | 1.06e-05 | 0.5%, 16 h |
| *pm2_80.37* | 556 | 52,061,452 | 1.07e-05 | 0.5%, 16 h |
| *pm2_81.329* | 602 | 52,061,452 | 1.16e-05 | 0.5%, 16 h |
| *pm2_90.93* | 753 | 52,061,452 | 1.45e-05 | 0.5%, 16 h |

*Cultivar contaminant

**Table S5.** Barley *Eceriferum-q* and *Pm2* alleles sequenced in this study.

| **Accession^1^** | **Accession name** | **Phenotype** | **Cultivar** | **EMS^7^ dose** | **SNV** | **Amino acid** |
| --- | --- | --- | --- | --- | --- | --- |
| NGB14659 | Wildtype | Waxed | Foma | 0 |  |  |
| NGB131725 | Wildtype | Waxed | Bonus | 0 |  |  |
| NGB14662 | Wildtype | Waxed | Kristina | 0 |  |  |
| NGB111182 | *eceriferum-q.295*^2^ | No wax | Foma | 0.12%, 25 h | C38T | A13V |
| NGB111184 | *eceriferum-q.297*^2^ | No wax | Foma | 0.25%, 72 h | T604A | W202R |
| NGB111197 | *eceriferum-q.310*^2^ | No wax | Foma | 0.17%, 24 h | G506A | S169N |
| NGB111214 | *eceriferum-q.327*^2^ | No wax | Foma | 0.25%, 5 h | G515A | G172D |
| NGB111221 | *eceriferum-q.334*^2,3^ | Semi waxed | Foma | 0.7%, 5 h |  |  |
| NGB111252 | *eceriferum-q.365*^2^ | No wax | Foma | 0.17%, 5 h | G515A | G172D |
| NGB112016 | *eceriferum-q.1128* | No wax | Kristina | 0.375%, 24 h | C1040T | P347L |
| NGB112113 | *eceriferum-q.1225* | No wax | Kristina | 0.91%, 5 h | G293A | S98N |
| NGB112171 | *eceriferum-q.1283* | No wax | Kristina | 0.65%, 5 h | G385A | E129K |
| NGB116826 | *eceriferum-q.1358* | No wax | Bonus | 1.29%, 5 h | G669A | W223* |
| NGB112288 | *eceriferum-q.1400* | No wax | Bonus | 0.65%, 5 h | C733T | P245S |
| NGB112555 | *eceriferum-q.1742* | No wax | Bonus | 0.65%, 5 h | G670A | V224M |
| CItr12632 | *pm2*_78_173^4^ | 87.3 ± 0.7^5^ | Allard^6^ | 0.5%, 16 h | G31A | G11R |
| CItr12632 | *pm2*_77_214^4^ | 87.4 ± 0.5^5^ | Allard^6^ | 0.5%, 16 h | C59T | S20F |
| CItr12632 | *pm2*_88_77^4^ | 81.7 ± 1.9^5^ | Allard^6^ | 0.5%, 16 h | G166A | V56M |
| CItr12632 | *pm2*_81_329^2^ | 80.7 ± 1.9^5^ | Allard^6^ | 0.5%, 16 h | C202T | Q68* |
| CItr12632 | *pm2*_78_56^2^ | 86.0 ± 1.6^5^ | Allard^6^ | 0.5%, 16 h | G256A | A86T |
| CItr12632 | *pm2*_90_93^2^ | 88.8 ± 0.7^5^ | Allard^6^ | 0.5%, 16 h | G256A | A86T |
| CItr12632 | *pm2*_80_355^2^ | 60.3 ± 2.7^5^ | Allard^6^ | 0.5%, 16 h | G499A, G973A | D167N, E325K |
| CItr12632 | *pm2*_79_241^4^ | 80.0 ± 1.0^5^ | Allard^6^ | 0.5%, 16 h | C751T | Q251* |
| CItr12632 | *pm2*_86_298^4^ | 89.7 ± 0.3^5^ | Allard^6^ | 0.5%, 16 h | G785A | W262* |
| CItr12632 | *pm2*_84_16^4^ | 84.0 ± 1.2^5^ | Allard^6^ | 0.5%, 16 h | C1264T | Q422* |
| CItr12632 | *pm2*_80_37^2^ | 86.3± 1.3^5^ | Allard^6^ | 0.5%, 16 h | C1576T | Q526* |
| CItr12632 | *pm2*_80_323^2^ | 81.7 ± 2.2^5^ | Allard^6^ | 0.5%, 16 h | G2048A | G683E |

^1^ NG = Nordgen accession, CItr12632 = USDA Germplasm Resource Information Network (GRIN) accession.

^2^ Sequenced by MutChromSeq then confirmed by Sanger sequencing.

^3^ *Eceriferum-q.334*, cultivar contaminant.

^4^ Sanger sequencing.

^5^ Percentage of the surface area of tested leaf segments infected (means of ten biological replicates ± SE).

^6^ Allard No. 52-1-1-17-1.

^7^ EMS = ethyl methanesulfonate.

**Table S6.** Reactions of the *Pm2* near-isogenic lines used in the present study after inoculation with 32 isolates of *Blumeria graminis f. sp. tritici.*

|  | ***Pm2* line** | |
| --- | --- | --- |
| ***Bgt* isolate** | **CI12632/8*Cc** | **Federation*4/Ulka** |
| 98430 | 95 ± 2.9 (S) | 87 ± 3.3 (S) |
| 98415 | 97 ± 3.3 (S) | 93 ± 6.7 (S) |
| 98411 | 87 ± 3.3 (S) | 90 ± 0.0 (S) |
| 98398 | 93 ± 3.3 (S) | 90 ± 0.0 (S) |
| 98250 | 87 ± 3.3 (S) | 90 ± 0.0 (S) |
| 98230 | 87 ± 3.3 (S) | 93 ± 3.3 (S) |
| 98229 | 100 ± 0.0 (S) | 100 ± 0.0 (S) |
| 98226 | 100 ± 0.0 (S) | 100 ± 0.0 (S) |
| 98013 | 100 ± 0.0 (S) | 100 ± 0.0 (S) |
| 97266 | 0 ± 0.0 (R) | 0 ± 0.0 (R) |
| 97251 | 100 ± 0.0 (S) | 100 ± 0.0 (S) |
| 97235 | 2 ± 1.7 (R) | 2 ± 1.7 (R) |
| 97223 | 100 ± 0.0 (S) | 100 ± 0.0 (S) |
| 97042 | 0 ± 0.0 (R) | 0 ± 0.0 (R) |
| 97028 | 87 ± 3.3 (S) | 90 ± 0.0 (S) |
| 97019 | 77 ± 3.3 (S) | 87 ± 3.3 (S) |
| 97011 | 0 ± 0.0 (R) | 0 ± 0.0 (R) |
| 96249 | 2 ± 1.7 (R) | 2 ± 1.7 (R) |
| 96244 | 90 ± 0.0 (S) | 93 ± 3.3 (S) |
| 96238 | 100 ± 0.0 (S) | 100 ± 0.0 (S) |
| 96236 | 100 ± 0.0 (S) | 93 ± 3.3 (S) |
| 96229 | 2 ± 1.7 (R) | 2 ± 1.7 (R) |
| 96224 | 0 ± 0.0 (R) | 0 ± 0.0 (R) |
| 96221 | 63 ± 3.3 (S) | 63 ± 3.3 (S) |
| 94204 | 67 ± 3.3 (S) | 67 ± 3.3 (S) |
| 94202 | 0 ± 0.0 (R) | 2 ± 1.7 (R) |
| 90033 | 3 ± 1.7 (R) | 2 ± 1.7 (R) |
| 10012 | 83 ± 3.3 (S) | 73 ± 3.3 (S) |
| 10005 | 93 ± 3.3 (S) | 93 ± 3.3 (S) |
| 10003 | 100 ± 0.0 (S) | 100 ± 0.0 (S) |
| 10001 | 97 ± 3.3 (S) | 83 ± 3.3 (S) |
| JIW2 | 100 ± 0.0 (S) | 100 ± 0.0 (S) |

Values refer to percentage of the surface area of tested leaf segments infected (means of three biological replicates ± SE). R, resistant. S, susceptible.

**Table S7.** Primers used in this study.

| **Primer** | **Sequence** | **Description** | **Function** |
| --- | --- | --- | --- |
| EF1-α Forward | TGAAGAAGGTCGGCTACAACCC |  | Barley control |
| EF1-α Reverse | CGTCCTTGGAGTTGGAAGCAAC |  | Barley control |
| *Eceriferum.q*_amp Forward | GTGACCTGAGGCCTGCATATTG |  | Amplification |
| *Eceriferum.q*_amp Reverse | CGTGGTGAATTGTGGCGACTAG |  | Amplification |
| *Eceriferum.q*_1 Forward | CTCCAGACGTGATCATAAC |  | Sequencing |
| *Eceriferum.q*_1 Reverse | ATCCGAGGATCATCGTTGC |  | Sequencing |
| *Eceriferum.q*_2 Forward | GCAAACCTAGCTTGTGC |  | Sequencing |
| *Eceriferum.q*_2 Reverse | CCACGTCCTACGATGACAC |  | Sequencing |
| ctg_17818_Federation*4/Ulka | CCCATGAGGGACGAGTCA | Federation*4/Ulka = A | Map *Pm2* |
| ctg_17818_Federation | CTCCCATGAGGGACGAGTCG | Federation = G | Map *Pm2* |
| ctg_17818_Common | GGGTTGCAACTGCGCTAGGGTT |  | Map *Pm2* |
| ctg_8619_Federation*4/Ulka | GGCCTTAGTCTACGAGTCTTCTT | Federation*4/Ulka = A | Map *Pm2* |
| ctg_8619_Federation | GGCCTTAGTCTACGAGTCTTCTG | Federation = C | Map *Pm2* |
| ctg_8619_Common | ACCCAACATGGGCTAACACACGTTA |  | Map *Pm2* |
| ctg_74401_Federation*4/Ulka | AACCTTAACAGATCTCGAAGACACATT | Federation*4/Ulka = T | Map *Pm2* |
| ctg_74401_Federation | CCTTAACAGATCTCGAAGACACATC | Federation = C | Map *Pm2* |
| ctg_74401_Common | CCGGTGTGAGGGCTCCGTTTTT |  | Map *Pm2* |
| ctg_81209_Federation*4/Ulka | AGAACCAGCGCTACTTGACCG | Federation*4/Ulka = G | Map *Pm2* |
| ctg_81209_Federation | AGAACCAGCGCTACTTGACCA | Federation = A | Map *Pm2* |
| ctg_81209_Common | GCTTAGCATATTGTGCGCTACGGAT |  | Map *Pm2* |
| ctg_184411_Federation*4/Ulka | GGAAAAGAGTCAGGCTGGGCAA | Federation*4/Ulka = T | Map *Pm2* |
| ctg_184411_Federation | GAAAAGAGTCAGGCTGGGCAG | Federation = C | Map *Pm2* |
| ctg_184411_Common | CAAGCAATCAAACTACCTTACAATGTCGTT |  | Map *Pm2* |
| ctg_81718_Federation*4/Ulka | AGTCTCTCGGAGATGCTCATAGA | Federation*4/Ulka = A | Map *Pm2* |
| ctg_81718_Federation | GTCTCTCGGAGATGCTCATAGG | Federation = G | Map *Pm2* |
| ctg_81718_Common | CATATGAACACACGCACACACACTCTT |  | Map *Pm2* |
| ctg_92428_Federation*4/Ulka | TATCAGTTTCCTTTGTAGCCACG | Federation*4/Ulka = G | Map *Pm2* |
| ctg_92428_Federation | CCTTATCAGTTTCCTTTGTAGCCACA | Federation = A | Map *Pm2* |
| ctg_92428_Common | AAAGGTAGTTGCTATTGGTATGGCATCAT |  | Map *Pm2* |
| *Xcfd81*-F | TATCCCCAATCCCCTCTTTC | Forward | Map *Pm2* |
| *Xcfd81*-R | GTCAATTGTGGCTTGTCCCT | Reverse | Map *Pm2* |
| JS303 | GAGTTGTAAAAGGAGAGTAATCG | Use with JS305 | Map 7526_1 |
| JS305 | AATGATAGCATGCATTTGGAG | Use with JS303 | Map 7526_1 |
| JS354 | AAGAGTCTCTGCCACATCGG | Use with JS355 | Map 7526_1 |
| JS355 | CAGCCATCGTTCAGGTAAGC | Use with JS354 | Map 7526_1 |
| JS320 | ACGATGATGTGAATCTTCCGTG | Use with JS305 | PCR 1, Fig S3 |
| JS305 | AATGATAGCATGCATTTGGAG | Use with JS320 | PCR 1, Fig S3 |
| JS350 | CCCTCCTCCTTGAAGAATCTGA | Use with JS313 | PCR 2, Fig S3 |
| JS313 | GCACAAACTCTACCCTGTTCC | Use with JS350 | PCR 2, Fig S3 |
| JS314 | TTTTCGCGGTATTGCTGGTG | Use with JS315 | Nested PCR |
| JS315 | ACCTCCTGTCATCGGTTCAC | Use with JS314 | Nested PCR |
| JS303 | GAGTTGTAAAAGGAGAGTAATCG |  | Sequence *Pm2* |
| JS305 | AATGATAGCATGCATTTGGAG |  | Sequence *Pm2* |
| JS317 | TCGGTTCACAGTCAGATCAGG |  | Sequence *Pm2* |
| JS321 | CTTGTTGATAGCAGGGCCAAA |  | Sequence *Pm2* |
| JS324 | CACATATCCAGGCGGTGTTC |  | Sequence *Pm2* |
| JS328 | GAACCACTACAACGGGACCT |  | Sequence *Pm2* |
| JS330 | ACCGGTGAATGTGCAAGAATAG |  | Sequence *Pm2* |
| JS342 | GTTGAGGACGATGAGAGATGC |  | Sequence *Pm2* |
| JS345 | ATGTTCTGCAGCGACCTGAA |  | Sequence *Pm2* |
| JS322 | ATATCCAGGCGGTGTTCGAT | Use with JS362 | 401 bp cDNA |
| JS362 | GCGATCATTGTCCATGAGCTG | Use with JS322 | 401 bp cDNA |

**Table S8.** Simulation results for likely number of contigs to be mutated (canonical SNPs only) simultaneously across a given number of compared mutants. A value less than 1 indicates that in all likelihood, there will be no false positives.

|  | **Wheat** | **Barley** |
| --- | --- | --- |
| Number of contigs post processing* | 77,136 | 204,389 |
| Probable number of contigs mutated for 1 mutant | 1,518 | 1,722 |
| Probable number of contigs mutated for 2 mutants | 75 | 43 |
| Probable number of contigs mutated for 3 mutants | 7.6 | 2.4 |
| Probable number of contigs mutated for 4 mutants | 1.1 | 0.24 |
| Probable number of contigs mutated for 5 mutants | 0.22 | 0.04 |
| Probable number of contigs mutated for 6 mutants | 0.05 | 0.01 |

*Contigs <500 bp and/or with coverage <15 were removed.

**Table S9.** Mutation probability calculations for the functional mutable space in the barley *Eceriferum-q* and wheat *Pm2* genes.

|  | **GC mutation frequency** | **Contig average GC content** | **Mutable length** | **GC mutation probability in single mutant** | **Probability of contig being mutated in multiple mutants** |
| --- | --- | --- | --- | --- | --- |
| Barley  (*Eceriferum-q*) | 9.28e-06 | 44.9%  (SD = 9.1%) | 5,387 bp  (in 12 kb contig) | *p* = 0.05  (in 12 kb contig) | *p* = 0.05^11^ = 1 in 4 · 10^14^  (in 11 mutants)) |
| Wheat  (*Pm2*) | 2.43e-05 | 44.1%  (SD = 7.8) | 4,411 bp  (in 10 kb contig) | *p* = 0.11  (in 10 kb contig) | *p* = 0.11^12^ = 1 in 3 · 10^11^  (in 12 mutants) |

**Supplementary References**

1. Lundqvist U, von Wettstein D: **Induction of *Eceriferum* mutants in barley by ionizing radiations and chemical mutagens.** *Hereditas* 1962, **48:**342-+.

2. Lundqvis U, von Wettstein P, von Wettstein D: **Induction of Eceriferum mutants in barley by ionizing radiations and chemical mutagens.** *Hereditas-Genetiskt Arkiv* 1968, **59:**473-&.

3. Mayer IBGSCKFX, Waugh R, Brown JWS, Schulman A, Langridge P, Platzer M, Fincher GB, Muehlbauer GJ, Sato K, Close TJ, et al: **A physical, genetic and functional sequence assembly of the barley genome.** *Nature* 2012, **491:**711-716.

4. International Barley Genome Sequencing C, Mayer KF, Waugh R, Brown JW, Schulman A, Langridge P, Platzer M, Fincher GB, Muehlbauer GJ, Sato K, et al: **A physical, genetic and functional sequence assembly of the barley genome.** *Nature* 2012, **491:**711-716.

5. Colmsee C, Beier S, Himmelbach A, Schmutzer T, Stein N, Scholz U, Mascher M: **BARLEX - the Barley Draft Genome Explorer.** *Mol Plant* 2015, **8:**964-966.

6. Pallotta MA, Warner P, Fox RL, Kuchel SJ, Jeffreies SJ, Langridge P: **Marker assisted wheat breeding in the southern region of Australia.** *Proceedings of the Tenth International Wheat Genetics Symposium, Paestum, Italy* 2003**:**789-791.

7. McIntosh RA, Baker EP: **Cytogenetic studies in wheat IV. Chromosome location and linkage studies involving the *Pm2* locus for powdery mildew resistance.** *Euphytica* 1970, **19:**71-77.

8. Pugsley AT, Carter MV: **The resistance of twelve varieties of *Triticum vulgare* to *Erysiphe graminis tritici*.** *Aust J Biol Sci* 1953, **6:**335-346.

9. Nyquist WE: **Inheritance of Powdery Mildew resistance in hybrids involving a common wheat strain derived from *Triticum timopheevi*.** *Crop Sci* 1963, **3:**40-43.

10. Briggle LW: **Three loci in wheat involving resistance to *Erysiphe graminis* f. sp. *tritici*.** *Crop Sci* 1966, **6**.

11. Ma P, Xu H, Xu Y, Li L, Qie Y, Luo Q, Zhang X, Li X, Zhou Y, An D: **Molecular mapping of a new powdery mildew resistance gene *Pm2b* in Chinese breeding line KM2939.** *Theor Appl Genet* 2015, **128:**613-622.

12. Xu H, Yi Y, Ma P, Qie Y, Fu X, Xu Y, Zhang X, An D: **Molecular tagging of a new broad-spectrum powdery mildew resistance allele *Pm2c* in Chinese wheat landrace Niaomai.** *Theor Appl Genet* 2015, **128:**2077-2084.

13. Stein N, Herren G, Keller B: **A new DNA extraction method for high-throughput marker analysis in a large-genome species such as *Triticum aestivum*.** *Plant Breeding* 2001, **120:**354-356.

14. Winzeler M, Streckeisen P, Fried PM: **Virulence Analysis of the Wheat Powdery Mildew Population in Switzerland between 1980 and 1989.** *Integrated Control of Cereal Mildews : Virulence Patterns and Their Change* 1991**:**15-21.

15. Lutz J, Limpert E, Bartos P, Zeller FJ: **Identification of Powdery Mildew resistance genes in common wheat (*Triticum aestivum* L.).** *Plant Breeding* 1992, **108:**33-39.

16. Kaur N, Street K, Mackay M, Yahiaoui N, Keller B: **Molecular approaches for characterization and use of natural disease resistance in wheat.** *Eur J Plant Pathol* 2008, **121:**387-397.

17. Shatalina M, Wicker T, Buchmann JP, Oberhaensli S, Simkova H, Dolezel J, Keller B: **Genotype-specific SNP map based on whole chromosome 3B sequence information from wheat cultivars Arina and Forno.** *Plant Biotechnol J* 2013, **11:**23-32.

18. International Wheat Genome Sequencing C: **A chromosome-based draft sequence of the hexaploid bread wheat (*Triticum aestivum*) genome.** *Science* 2014, **345:**1251788.

19. Qiu YC, Sun XL, Zhou RH, Kong XY, Zhang SS, Jia JZ: **Identification of microsatellite markers linked to powdery mildew resistance gene *Pm2* in wheat.** *Cereal Research Communications* 2006, **34:**1267-1273.

20. Li G-Q, Fang T-L, Zhu J, Gao L-L, Li S, Xie C-J: **Molecular Identification of a Powdery Mildew resistance gene from common wheat cultivar Brock.** *Acta Agron Sin* 2009, **35:**1613-1619.

21. Wang SC, Wong DB, Forrest K, Allen A, Chao SM, Huang BE, Maccaferri M, Salvi S, Milner SG, Cattivelli L, et al: **Characterization of polyploid wheat genomic diversity using a high-density 90,000 single nucleotide polymorphism array.** *Plant Biotechnol J* 2014, **12:**787-796.

22. Kosambi DD: **The estimation of map distances from recombination values.** *Annals of Eugenics* 1943, **12:**172-175.

23. Voorrips RE: **MapChart: software for the graphical presentation of linkage maps and QTLs.** *J Hered* 2002, **93:**77-78.

24. Somers DJ, Isaac P, Edwards K: **A high-density microsatellite consensus map for bread wheat (*Triticum aestivum* L.).** *Theor Appl Genet* 2004, **109:**1105-1114.

25. Henry IM, Nagalakshmi U, Lieberman MC, Ngo KJ, Krasileva KV, Vasquez-Gross H, Akhunova A, Akhunov E, Dubcovsky J, Tai TH, Comai L: **Efficient Genome-Wide Detection and Cataloging of EMS-Induced Mutations Using Exome Capture and Next-Generation Sequencing.** *Plant Cell* 2014, **26:**1382-1397.

26. Uauy C, Paraiso F, Colasuonno P, Tran RK, Tsai H, Berardi S, Comai L, Dubcovsky J: **A modified TILLING approach to detect induced mutations in tetraploid and hexaploid wheat.** *BMC Plant Biol* 2009, **9:**115.

27. Farrell A, Coleman BI, Benenati B, Brown KM, Blader IJ, Marth GT, Gubbels MJ: **Whole genome profiling of spontaneous and chemically induced mutations in Toxoplasma gondii.** *BMC Genomics* 2014, **15:**354.

28. Shirasawa K, Hirakawa H, Nunome T, Tabata S, Isobe S: **Genome-wide survey of artificial mutations induced by ethyl methanesulfonate and gamma rays in tomato.** *Plant Biotechnol J* 2016, **14:**51-60.

29. Lindner H, Kessler SA, Muller LM, Shimosato-Asano H, Boisson-Dernier A, Grossniklaus U: **TURAN and EVAN mediate pollen tube reception in Arabidopsis Synergids through protein glycosylation.** *PLoS Biol* 2015, **13:**e1002139.
